# Elevated procalcitonin levels in primary hepatic neuroendocrine carcinoma: Case report and literature review

**DOI:** 10.1097/MD.0000000000021210

**Published:** 2020-07-31

**Authors:** Xiangjun Han, Hongshan Zhong, Duo Hong, Chenguang Li, Hongying Su, Ke Xu

**Affiliations:** Department of Interventional Radiology, the First Hospital of China Medical University, Shenyang, Liaoning, P.R. China.

**Keywords:** biomarker, liver, neuroendocrine carcinoma, procalcitonin

## Abstract

**Rationale:**

Procalcitonin (PCT) has been identified as a tumor biomarker in medullary thyroid carcinoma. Other neuroendocrine carcinomas with elevated PCT levels are relatively rare, and are mainly reported in the lung, digestive tract, and pancreas. No studies in the literature have reported a case of primary hepatic carcinoma complicated with unexpectedly elevated PCT levels.

**Patient concerns:**

A 78-year-old man with persistent fatigue and mild fever was complicated with an extremely high PCT level. Radiological examination revealed a single hypodense lesion in the left lobe of the liver with a “rapid enhancement and rapid washout” pattern. Pathological analysis showed a poorly differentiated neuroendocrine carcinoma (grade 3) with multiple genetic mutations.

**Diagnosis:**

Primary hepatic neuroendocrine carcinoma.

**Interventions:**

The patient received antibiotic therapy and subsequent transcatheter hepatic arterial chemoembolization; a PCT assessment and computed tomography were performed during the follow-up.

**Outcomes:**

The PCT level did not decline after antibiotic therapy but greatly declined in response to effective transcatheter hepatic arterial chemoembolization. The patient survived and is still being followed up.

**Lessons:**

An extremely elevated PCT level may raise a suspicion of a neuroendocrine carcinoma and plays an indicative role as a biomarker during therapy.

## 1 Introduction

Procalcitonin (PCT) is a precursor of calcitonin that is mainly secreted from thyroid cells,^[1]^ and it is rapidly produced in response to microbial toxins and inflammatory mediators. Due to its high specificity and sensitivity, PCT was established as an infectious biomarker several decades ago and is mainly used to diagnose and evaluate bacterial infections in clinical practice.^[2,3]^ PCT also plays an indicative role as a diagnostic tumor biomarker in medullary thyroid carcinoma.^[4]^ A high PCT level indicates a poor prognosis. This finding has been elucidated in detail in many previous studies.^[4–6]^ Currently, an elevated PCT level has also been reported in other neuroendocrine tumors, including tumors in the lung, digestive tract, and pancreas.^[7–9]^ However, to the best of our knowledge, there are no previous reports of primary hepatic carcinoma complicated with unexpectedly elevated PCT. This brief report presents a 78-year-old man with a pathologically identified primary hepatic neuroendocrine carcinoma with an extremely elevated PCT level.

## 2 Case report

**Figure 1:**
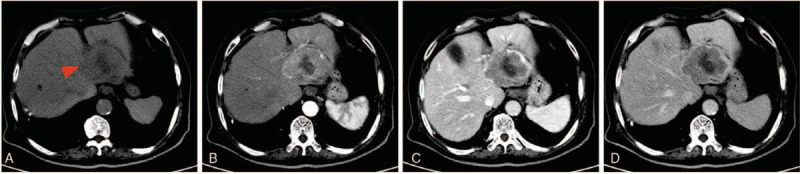
Enhanced CT images of primary hepatic neuroendocrine carcinomas. The enhanced CT demonstrated a “rapid enhancement and rapid washout” pattern. (A) A 6 × 5 cm hypodense lesion with necrosis in the center of the left lobe of the liver (red arrow). (B) The lesion showed rapid enhancement in the arterial phase, and the relatively lower density that followed indicates a quick washout in the portal phase (C) and delayed phase (D). CT = computed tomography.

**Figure 2:**
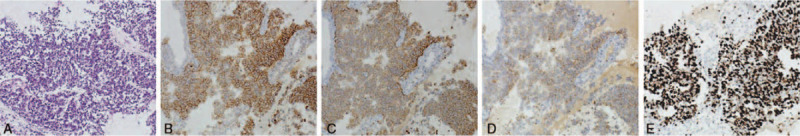
Pathological expression of a primary hepatic neuroendocrine carcinoma. Histological analysis revealed atypical cells with enlarged nuclei after hematoxylin-eosin staining of the specimens obtained by biopsy (A). Immunohistochemistry revealed the positive expression of synaptophysin (B), chromogranin A (C), and CD 56 (D). The diffuse expression of Ki-67 suggested poorly differentiated carcinoma and a high degree of malignancy (E).

A 78-year-old Chinese man presented to our hospital with persistent fatigue and a mild fever of 38.5 °C. He had no history of abdominal pain, chronic hepatitis virus infection, or excessive drinking. The physical examination results were unremarkable. His radiological examination with computed tomography (CT) revealed a single hypodense lesion in the left lobe of the liver with a “rapid enhancement and rapid washout” pattern (Fig. 1). Laboratory results showed normal hepatic function, renal function, leukocyte counts, and alpha-fetoprotein (AFP) levels. Unexpectedly, his serum PCT level was extremely high at 78.96 ng/mL (reference value <0.05 ng/mL). He received 3 days of intravenous antibiotics to treat a possible infection, and his temperature became normal, but the PCT level remained high. Subsequently, a percutaneous biopsy of the lesion was conducted under CT guidance. Histological analysis revealed the positive expression of synaptophysin, chromogranin A, CD 56, and Ki-67 (labeling index = 90%) (Fig. 2). Therefore, the patient was pathologically diagnosed with poorly differentiated primary hepatic neuroendocrine carcinoma (grade 3). Genetic analysis demonstrated that the tumor had multiple mutations of RB1 (exon 19, p.K652fs), TP53 (exon 5, p.R175H), BRAF (exon 11, p.G466R), MAP2K2 (exon 3, p.Y134H), and NF1 (exon 16, p.L585fs; exon 46, p.Y2285fs).

**Figure 3:**
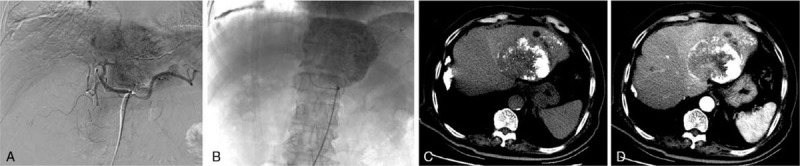
TACE and therapeutic efficacy evaluation of primary hepatic neuroendocrine carcinoma. Digital subtraction angiography demonstrated a hypervascular nature (A), and the patient underwent TACE with lipiodol and cisplatin (B). A therapeutic efficacy evaluation was conducted within one month and revealed a partial response according to the mRECIST standard criteria (C in the arterial phase and D in the portal phase). mRECIST = modified Response Evaluation Criteria in Solid Tumors (mRECIST); TACE = transcatheter hepatic arterial chemoembolization.

**Figure 4:**
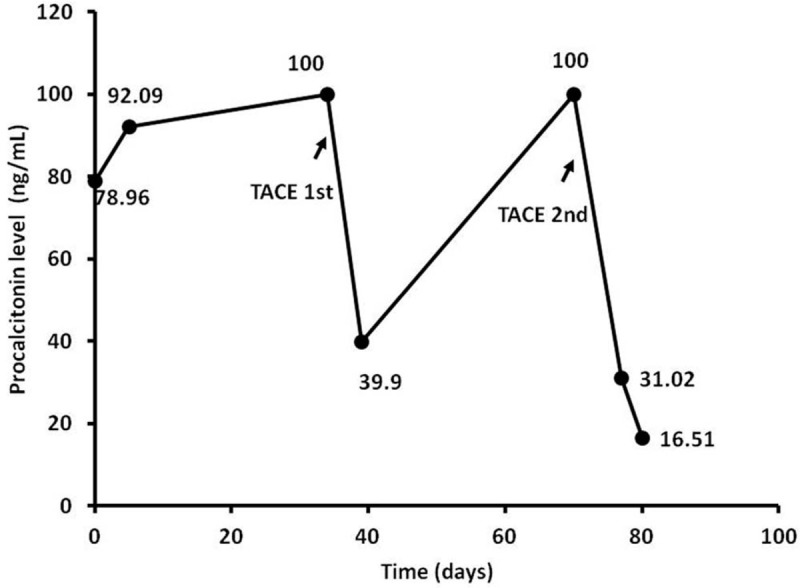
PCT response to antitumor therapy in primary hepatic neuroendocrine carcinoma. The PCT levels greatly declined after each effective TACE procedure performed on day 35 and 70 after admission. PCT = procalcitonin, TACE = transcatheter hepatic arterial chemoembolization.

The patient underwent transcatheter hepatic arterial chemoembolization (TACE) with lipiodol and cisplatin (Fig. 3A and B), and his PCT level greatly decreased from 100 to 39.9 ng/mL. The therapeutic efficacy evaluation within 1 month revealed a partial response according to the modified Response Evaluation Criteria in Solid Tumors (mRECIST) criteria (Fig. 3C and D). He underwent a second TACE procedure, and his PCT level eventually decreased to 16.51 ng/mL (Fig. 4). The patient survived for 6 months after the tumor diagnosis and underwent regular follow-up evaluations.

## 3 Consent for publication

Written informed consent was obtained from the patient for publication of this case report and accompanying images. Institutional review board approval was not required at our institution for case report.

## 4 Discussion

PCT, a peptide consisting of 116 amino acids, has been identified as a sensitive and specific serum biomarker of bacterial infections for several decades.^[10]^ This protein is mainly released from thyroid cells and partially released from neuroendocrine cells that are mostly located in the lung and digestive system. Thus, elevated PCT levels have been indicated to be a tumor biomarker for medullary thyroid cancers and have been found in some neuroendocrine carcinomas.^[5,8]^ Due to the clinical unfamiliarity and low incidence of elevated PCT levels in other types of tumors, there is no previous evidence of an extremely high PCT level in liver neuroendocrine carcinoma. This case report demonstrates an extremely elevated PCT level in a primary hepatic neuroendocrine carcinoma, and the most interesting point is that the PCT level significantly declined after each round of effective antitumor therapy.

To investigate the association between tumors and elevated PCT levels, we conducted a literature review on this topic. We searched the PubMed database using the key words “procalcitonin” AND (“cancer” OR “tumor” OR “carcinoma”). Five hundred and eighty-three relevant publications were identified, and 45 papers focused on PCT as a tumor biomarker in various carcinomas. Of those papers, 35 focused on medullary thyroid carcinoma, 6 papers focused on lung cancer, 2 papers focused on digestive system cancer, and 2 papers focused on pancreatic cancer. In total, there were 4 reports of tumors with an extremely elevated PCT level in the literature. All tumors, including esophageal, pulmonary, and pancreatic tumors were neuroendocrine carcinomas with liver metastasis, and PCT levels ranged from 20.1 to 927.0 ng/mL. Of these patients, 1 patient received effective antitumor therapy, and the PCT level correspondingly decreased.^[7–9,11]^ Chen et al^[12]^ described 155 digestive neuroendocrine tumors, and elevated serum PCT values were found in 40.6% of patients who were sensitive to antitumor therapies. TACE has been established as an effective method to treat hepatic carcinoma.^[13]^ In our case, the elevated PCT level declined after 2 TACE procedures, which was consistent with the conclusion from previous reports. These collective data suggest that an obviously elevated PCT level and sensitivity to antitumor therapy indicate a possible role of PCT as a tumor biomarker for neuroendocrine carcinoma.

Patout et al^[14]^ revealed that a PCT level over 0.15 ng/mL was significantly associated with the presence of neuroendocrine cells in pulmonary tumors, but the sensitivity of 63.8% was not sufficiently high. Due to the limited data on the association between PCT levels and neuroendocrine carcinoma, an appropriate cutoff value for PCT and its corresponding sensitivity and specificity as a tumor diagnostic biomarker need to be further explored in the future. Several studies have shown that neuroendocrine carcinoma with liver metastasis usually has a higher PCT level.^[14,15]^ Previous studies have illustrated that 4 patients with obviously elevated PCT levels had similar conditions, and they all had liver metastasis. Our patient, who had a primary hepatic neuroendocrine carcinoma, also had an abnormally elevated PCT level. From our perspective, the abnormal elevation of PCT in these patients may be attributed to several factors. First, the neuroendocrine nature of the tumor cells will produce PCT that enters the serum for circulation. Second, the large number of neuroendocrine cells and Kupffer cells in the liver may play a partial role. Future studies are needed to elaborate the underlying mechanism.

This case report also has some limitations. First, the retrospective nature of this presentation and limited sample size may produce possible bias. Second, most cases of tumors with unexpectedly high PCT levels in the literature are medullary thyroid carcinomas, and PCT levels should be investigated in other neuroendocrine carcinomas in the future. Third, genetic analysis revealed that the tumor had multiple mutations, and targeted chemotherapy or immunotherapy should be evaluated in the future.

In conclusion, this case presents a primary hepatic neuroendocrine carcinoma complicated with an unexpectedly high PCT level, and the elevated PCT level was positively associated with the tumor status. This finding suggests the potentially indicative role of serum PCT in tumor patients, and an extremely elevated serum PCT level may raise a suspicion of neuroendocrine carcinoma as a diagnosis if no evidence of infection is found.

## Acknowledgments

The authors thank Dr. Yang Han in our institution for pathological analysis, and also thank Dr. Huina Wang, Dr. Jing Zhang, and Dr. Shanbo Cao in AcornMed Biotechnology Company for genetic analysis.

## Author contributions

**Conceptualization:** Hongying Su, Ke Xu.

**Data collection and analysis:** Xiangjun Han, Chenguang Li.

**Investigation:** Duo Hong, Chenguang Li.

**Manuscript writing:** Xiangjun Han, Hongshan Zhong.

**Review:** Hongshan Zhong.
